# Modeling and Control of a Cable-Driven Rotary Series Elastic Actuator for an Upper Limb Rehabilitation Robot

**DOI:** 10.3389/fnbot.2020.00013

**Published:** 2020-02-25

**Authors:** Qiang Zhang, Dingyang Sun, Wei Qian, Xiaohui Xiao, Zhao Guo

**Affiliations:** ^1^School of Power and Mechanical Engineering, Wuhan University, Wuhan, China; ^2^UNC/NCSU Joint Department of Biomedical Engineering, NC State University, Raleigh, NC, United States; ^3^Wuhan University Shenzhen Research Institute, Shenzhen, China

**Keywords:** series elastic actuator (SEA), rehabilitation robot, bowden cable, torque control, impedance control, disturbance observer (DOB)

## Abstract

This paper focuses on the design, modeling, and control of a novel remote actuation, including a compact rotary series elastic actuator (SEA) and Bowden cable. This kind of remote actuation is used for an upper limb rehabilitation robot (ULRR) with four powered degrees of freedom (DOFs). The SEA mainly consists of a DC motor with planetary gearheads, inner/outer sleeves, and eight linearly translational springs. The key innovations include (1) an encoder for direct spring displacement measurement, which can be used to calculate the output torque of SEA equivalently, (2) the embedded springs can absorb the negative impact of backlash on SEA control performance, (3) and the Bowden cable enables long-distance actuation and reduces the bulky structure on the robotic joint. In modeling of this actuation, the SEA's stiffness coefficient, the dynamics of the SEA, and the force transmission of the Bowden cable are considered for computing the inputs on each powered joint of the robot. Then, both torque and impedance controllers consisting of proportional-derivative (PD) feedback, disturbance observer (DOB), and feedforward compensation terms are developed. Simulation and experimental results verify the performance of these controllers. The preliminary results show that this new kind of actuation can not only implement stable and friendly actuation over a long distance but also be customized to meet the requirements of other robotic system design.

## Introduction

People with neurological disorders, such as stroke and spinal cord injury (SCI), usually have weakened function or dysfunction on upper limbs or lower limbs, which have significantly impeded the normal activities of daily living (ADLs) (Mackay, 2004). In recent years, much attention has been paid toward exoskeleton rehabilitation robots or devices. The most existing rehabilitation training devices introduce rigid actuators on active joints as the power generator, and they contribute to achieving more precise position movement and easier trajectory tracking control, as well as high response frequency (Ham et al., 2009; Kim and Bae, 2017). However, these robotic rehabilitation devices with rigid actuators have bulky structure and low back-drivability, which causes direct physical interaction with wearers when unexpected external impacts occur. Therefore, the interaction adaptability, safety, and robustness of rehabilitation devices with rigid actuators are significantly limited. Compared with rigid actuators, compliance is a typical characteristic of elastic actuators, and it has been introduced to guarantee the safety and comfort functionalities between human and robotic devices. The compliant actuators have several unique properties, such as low output impedance, passive mechanical energy storage and release (Zhang et al., 2015; Sun et al., 2017, 2018a,b). Instead of using force/torque sensor on a human joint, compliant actuators can be used to measure interaction forces directly (Yu et al., 2015; Pan et al., 2018b), which can be easily extended to estimate human motion intention for achieving assist-as-needed control strategy. Compared to the neuromuscular signals in Zhang et al. (2019a,b, 2020), force/torque can be acquired without complex signal processing, and it is much easier to achieve intention control in real-time.

**Figure 1:**
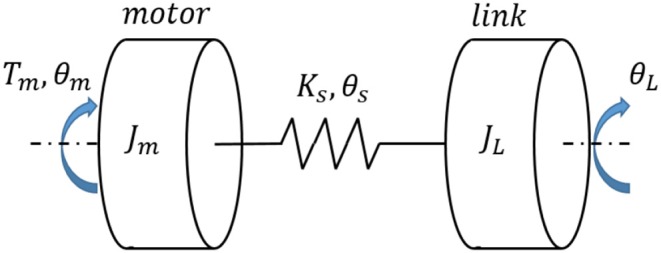
Schematic of the SEA concept.

One typical category of compliant actuators is the series elastic actuator (SEA), which includes a servo motor, translational or torsional springs, and an output mechanism. In general, the configuration of an SEA is shown in Figure 1, where *J*_*m*_ and *J*_*L*_ are the rotational inertia of the motor with gearheads and the output link, *T*_*m*_ is the motor's input torque, θ_*m*_ and θ_*L*_ are the angular position of the motor side and the link side. The spring's stiffness coefficient and angular deflection are represented as *K*_*s*_ and θ_*s*_, respectively.

SEAs have been developed for upper or lower limb rehabilitation devices (Kong et al., 2012; Wang et al., 2015). For example, Pratt et al. (2004) presented a linear SEA based on a linear spring coupled to a ball screw for knee exoskeleton design. A rotary SEA was proposed by Kong et al. (2009) to assist a lower limb movement. This kind of SEA included a torsional spring and two rotary potentiometers detecting the output position of the shaft and the deformation of the torsional spring. Accoto et al. (2013) presented an SEA for a lower limb exoskeleton-type robot. In this SEA, a novel torsional spring was implemented together with a set of bevel gears to handle the heavily loaded conditions. In actual application, torsional springs supporting large output torques are usually stiff, which results in lower torque control accuracy. In Carpino et al. (2012), the torsional spring constant for gait assistance usually reached the range from 100 to 300 N·m/rad, which generated the maximum torque with the range from 10 to 100 N·m. Yu et al. (2013) designed a compact compliant SEA, which could achieve reasonable force tracking at both low and high force range by using a set of translational springs and one torsional spring. Zhang and Collins (2017) found that the optimal passive stiffness matches the slope of the desired torque-angle relationship through walking experiments; therefore, they confirmed that the optimal passive stiffness benefits for lower-limb exoskeleton design.

In terms of the force/torque control of SEA, Pratt and Williamson (1995) proposed a linear compensator system to control the motor current with spring force feedback to guarantee an adequate torque. In Pan et al. (2018a), a second-order sliding mode control (SMC) law was proposed to guarantee the semi-global exponential stability of the robot dynamics. Most SEA controller design was a dynamic model-based approach; however, external disturbances and unexpected resonances could deteriorate the tracking performance or even cause instability. In Wang et al. (2019), by designing a sliding surface, a generalized proportional integral observer (GPIO)-based SMC was developed to track the desired trajectory while estimating the time-varying disturbance. A robust regulator for Markovian jump linear systems was proposed for SEA to guarantee the force control robustness in Jutinico et al. (2017). In Dos Santos and Siqueira (2014), the desired pole positions for an adaptive controller were determined in consideration of the disturbances on ideal torque source behavior. Another major problem in SEA control is the unknown load dynamics. A linear PID controller was formulated into a three-time-scale singular perturbation formula in Pan et al. (2019), and the advantages included simple structure and robustness for external disturbances and parameter variations. Paine et al. (2014) came up with the use of disturbance observers (DOB), and in that work, the controller was a state feedback controller with an integrator; also, the online parameter estimation was utilized to improve performance.

Although recently upper limb rehabilitation robotic devices have gained significant achievements, there are some drawbacks. Some rehabilitation devices are too large, heavy, and complex for medical and clinical applications because the actuators are usually installed precisely on the rotation joint or limb frames, which makes the joints heavier, more bulky, and stationary. In this work, our emphasis is to develop a novel kind of remote actuation, including a modular SEA with compactness and easy installation for an upper limb rehabilitation robot (ULRR). Besides, one challenge of this remote actuation is the accurate torque measurement on each degree of freedom (DOF) of ULRR. The installation of a torque sensor on each DOF is not practical due to space and mass limitation. Therefore, one sensorless approach is desired to compute the torque on each DOF. Once the transition model from the DC motor to each joint is known, torque on each joint DOF can be controlled by a specific controller. The essential inventions in this paper include: (1) only one rotary encoder is involved in measuring the spring's deflection based on the newly designed structure, (2) Bowden cable is utilized to implement remote actuation function and effectively reduce the weight of the wearable device, (3) torque controller consisting of DOB, feedforward friction and movement compensation is robust for unknown disturbance.

This paper is organized as follows: section Descriptions of Rotary SEA and ULRR presents the mechanical design description of the proposed SEA and its implementation on the ULRR. The kinetic model of the remote actuated system is described in section Modeling of the Remote Actuation, including the SEA's rotary stiffness coefficient, the Bowden cable's transmission model, and the SEA's dynamic model. Section Controller Design presents the torque and impedance controllers of this compliant actuator. Section Simulation Results presents the experimental results on the elbow joint by the remote actuation method. Section Experimental Results and Discussions presents conclusions and future work.

## Descriptions of Rotary SEA and ULRR

### Compact Rotary SEA

**Figure 2:**
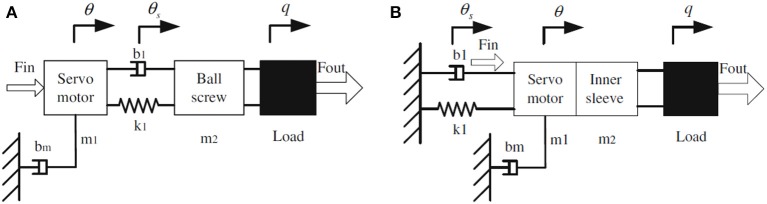
Structural configuration of traditional SEA and proposed SEA. **(A)** Pratt's general model. **(B)** Modified model.

Existing SEAs usually put elastic elements, such as linear translational or torsional springs, between the servo motor and outer load, and the output torque can be determined by controlling the deflection of the springs. In general, the SEA's servo motor housing is stationary, and its output side is away from the servo motor. Take the SEA proposed by Pratt et al. (2004) as an example, and its mechanical configuration is shown in Figure 2A. Although the rotation inertia is small in this design (only motor shaft, reducer shaft, and gears in reducer), the series components result in a longer structure. Also, due to the difficulty of directly measuring spring deflection, two encoders are needed. In this work, to address these two problems above, a novel structural arrangement of rotary SEA is proposed, as shown in Figure 2B.

**Figure 3:**
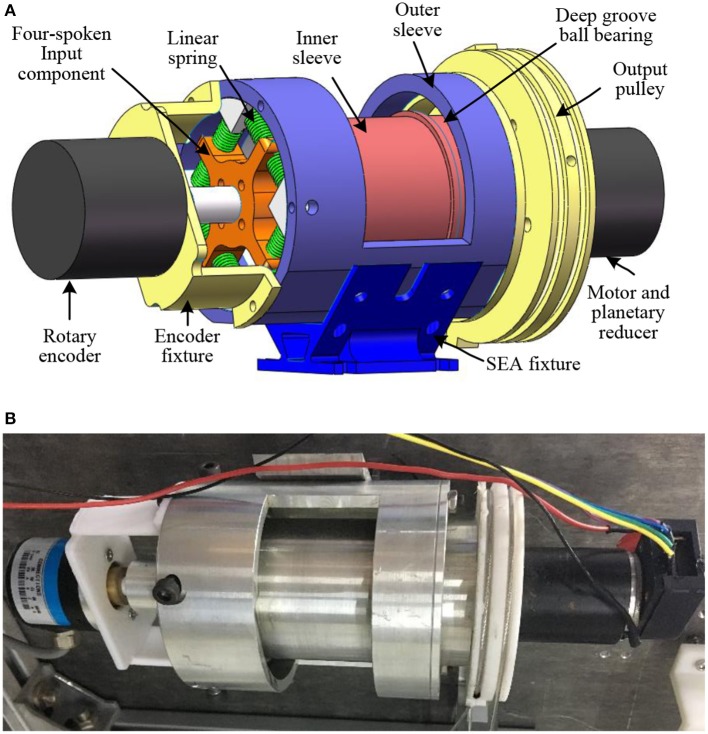
The prototype of the rotary SEA. **(A)** The CAD model for the rotary SEA built in SolidWorks. **(B)** The assembled prototype by using machined components.

Compared to Figure 2A, in the newly proposed structure, the position of the elastic element is changed from middle to the left, and the servo motor with reducer, inner sleeve, and pulley winded with steel cable are connected. The symbolic definition in Figure 2 will be explained in the modeling and controller design section. Figure 3 shows the 3-D CAD model and the machined SEA's prototype. There are two cylinder layers in this design, where the main components are the outer sleeve and inner sleeve, respectively. The gap on the outer sleeve is designed for the easy installation of deep groove ball bearings.

Our design of the novel SEA is based on the concept of closed-loop circuity, which can reduce the length of the traditional structure. As shown in Figure 3, the main components in the SEA include: (a) DC servo motor with planetary gear reducer, (b) rotary encoder, (c) four-spoke module, (d) linear springs, (e) deep groove ball bearings, (f) output pulley, (g) inner sleeve, and (h) outer sleeve. Here, it is assumed that the front side of the SEA is the rotary encoder, and the end side is the output pulley. Overall, the entire length of the SEA is 140 mm (plus the encoder on the front side), the maximum diameter is 80 mm, and the weight is 0.85 kg. There are two separate groove channels on the output pulley along its circumferential surface, where a pair of transmission Bowden cables are winded and fixed onto the two groove channels.

The power of this SEA comes from the electrical DC motor, whose output shaft is fixed with (c). Between (c) and (h), there are eight uniform components (d). The housing of the motor and reducer is installed coaxially with (g) through screws, and (g) is connected with (f) through geometric constrains. Two (e) are utilized to guarantee the coaxial property and smooth rotation motion between (g) and (h). The SEA can be regarded as a closed-loop kinematic chain in series. Moreover, when it works, the torque generated by the servo motor is transmitted to the joint through Bowden cables. Also, when external load occurs on the joint, the external torque is sent back to the SEA through Bowden cables. The torque loaded on the front side of the inner sleeve is equal to the torque generated by the linear springs' compression, and by measuring linear springs' deflection θ_*s*_, which is multiplied by the rotation stiffness coefficient, then the input torque of the inner sleeve is obtained. By subtracting the torque consumed by the inner sleeve, the output torque of the SEA on the output pulley is obtained.

### ULRR Actuated by SEA

**Figure 4:**
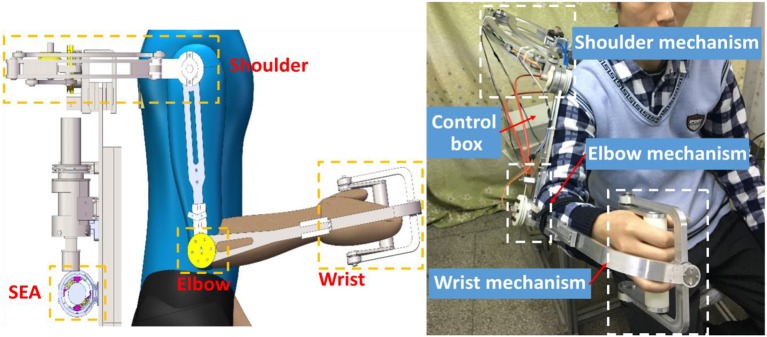
Physical prototype of upper limb rehabilitation robot (ULRR).

A lightweight, compliant, and power-efficient ULRR that can fulfill the task of upper limb motion assistance has been proposed, as shown in Figure 4. The hardware of ULRR mainly consists of machine frame, SEAs, embedded controller NI Single-Board RIO (sbRIO) 9637, DC motor drivers, and a PC, as shown in Figure 4. The structures of the upper arm and forearm are designed to be length-adjustable to satisfy the length requirements from different wearers with consideration of the ergonomics and biomechanics of the wearers.

This upper limb exoskeleton robotic device has four active DOFs, including three on the shoulder and one on the elbow. They are shoulder flexion/extension, adduction/abduction, internal/external rotation, and elbow flexion/extension, respectively. In the meanwhile, there are two passive DOFs on the wrist for its flexion/extension and supination/pronation rotation. For safety, mechanical stop constraints are designed for each active DOF on the extreme ends of the available range of motion. A special six-link structure is designed for the DOF of internal/external rotation on the shoulder, which increases the motion space of the ULRR as well as avoids the motion interference with wearers. When the shoulder joint works, three central axes from these three DOFs intersect on the same point, which is the same as the should ball joint center. This design can guarantee the motion alignment of the shoulder joint between the wearer and the robotic device. Each active DOF is powered by one SEA through a pair of Bowden cables. In order to reduce the weight and rotational inertia of each active DOF, instead of putting the SEAs on the links of the ULRR, the SEAs are installed on the back of the subject, away from joints and links. Between each SEA and joint DOF, there is a pair of Bowden cables delivering energy and motion for clockwise and counterclockwise rotation (Veneman et al., 2006). Due to the different power consumption for shoulder DOFs and elbow DOF, the DC motor's continuous maximum torques are not uniform. For the three DOFs on the shoulder, SEAs can provide maximum assistive torque 20 Nm, while for the elbow joint, SEA can provide maximum assistive torque 10 Nm.

## Modeling of the Remote Actuation

### Rotary Stiffness Model of SEA

**Figure 5:**
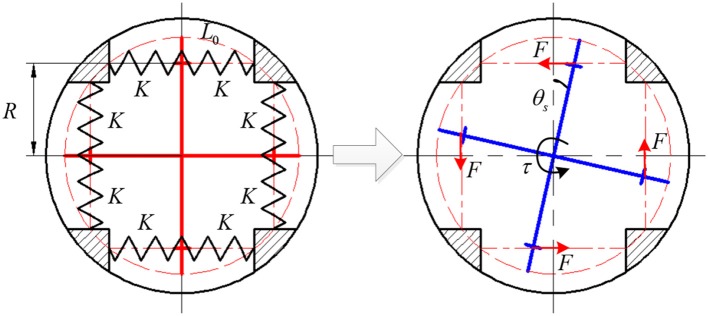
The compression of linear translational springs in SEA.

**Figure 6:**
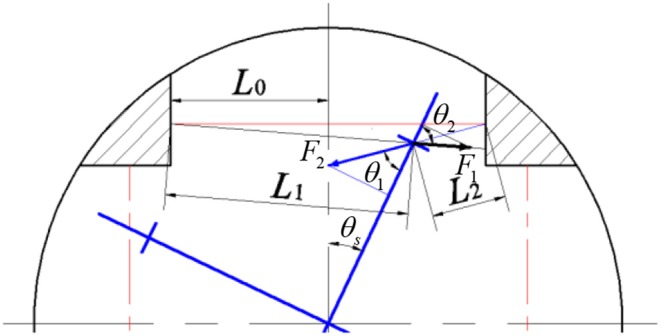
Antagonistic compression of one pair springs during SEA working.

The eight translational springs are the elastic module in the proposed SEA, the transformation from springs' linear compression stiffness to SEA rotary stiffness is derived in this section, and it is vital for the SEA torque control. The approximate SEA rotary stiffness model was presented in our previous work (Zhang et al., 2017), where the output torque of SEA is linearly related to θ_*s*_. Here, to eliminate the approximation error and improve the accuracy of torque control, we build the precise non-linear rotary stiffness model. Figure 5 shows the working mechanism of the eight springs embedded in SEA, which experience a pre-compression that equals to half of the maximum allowable compression within the elastic limit. Furthermore, the geometry design in Figure 5 guarantees the eight springs are compressed all the time when SEA works at the available angular deflection range. Due to the compactness and mechanical restriction of the SEA, the available deflection range is limited from −10 to 10, which means there is no more springs compression when θ_*s*_ reaches −10 or 10. No matter the four-spoke component rotates (clockwise or counterclockwise) relative to the outer sleeve, there are only four springs (right side or left side) experiencing more compression than the other side, as shown in Figure 6.

To derive the relationship between the angular deflection of the springs and the generated torque, the upper half of Figure 5 is enlarged, as shown in Figure 6. *L*_0_ is the length of springs after pre-compression, and the pre-compression length can be represented as Δ*x*. Then the original spring length *L* is given as

(1) $$\begin{array}{l} L=L_{0}+\Delta x. \end{array}$$

In Figure 6, the four-spoke component experiences an angular deflection of θ_*s*_. Moreover, the center axes of the spring pair will no longer stay along the primary axis. The solid red line represents the original axis of the spring pair without any angular deflection, the left and right solid black lines represent the center axis of the spring with smaller compression and the center axis of the spring with higher compression, respectively. Based on Cosine theorem, the current length of two springs in Figure 6 can be written as

(2) $$\begin{array}{l} L_{1}\\ =\sqrt{{\left(\frac{R}{cos\theta_{s}}-R\right)}^{2}+{\left(L_{0}+Rtan\theta_{s}\right)}^{2}-2\left(\frac{R}{cos\theta_{s}}-R\right)\left(L_{0}+Rtan\theta_{s}\right)sin\theta_{s}} \end{array}$$

(3) $$\begin{array}{l} L_{2}\\ =\sqrt{{\left(\frac{R}{cos\theta_{s}}-R\right)}^{2}+{\left(L_{0}-Rtan\theta_{s}\right)}^{2}+2\left(\frac{R}{cos\theta_{s}}-R\right)\left(L_{0}-Rtan\theta_{s}\right)sin\theta_{s}} \end{array}$$

where *R* represents the length of each four-spoke arm, *L*_1_ and *L*_2_ represent the length of the springs with smaller compression and higher compression, respectively. As mentioned before, the pair of springs are always compressed within the available angular deflection range. Thus, the new compression length of the two springs in Figure 6 now can be given as

(4) $$\begin{array}{l} \Delta x_{1}=L_{0}+\Delta x-L_{1} \end{array}$$

(5) $$\begin{array}{l} \Delta x_{2}=L_{0}+\Delta x-L_{2}. \end{array}$$

The directions of the anti-compression forces of each spring are on the same line along their center axes, respectively. Therefore, the force perpendicular to the four-spoke arm can be expressed as

(6) $$\begin{array}{l} F=F_{2}sin\theta_{1}-F_{1}sin\theta_{2} \end{array}$$

where

$$\begin{array}{l} {\;F}_{1}=K\Delta x_{1},\;F_{2}=K\Delta x_{2}\\ sin\theta_{1}=\frac{L_{0}-Rtan\theta_{s}}{L_{2}}cos\theta_{s}\\ sin\theta_{2}=\frac{L_{0}+Rtan\theta_{s}}{L_{1}}cos\theta_{s} \end{array}$$

where *K* is the spring stiffness constant, and the eight springs have the same *K*.

After the derivation of the above equations, the equivalent torque on the four-spoke component is given as

(7) $$\begin{array}{l} T_{total}=4KR\left(\Delta x_{2}sin\theta_{1}-\Delta x_{1}sin\theta_{2}\right). \end{array}$$

Then after taking the partial derivative of Equation (7) concerning θ_*s*_, the rotary stiffness coefficient of the four-spoke component can be expressed as the following non-linear function

(8) $$\begin{array}{l} K_{A}\left(\theta_{s}\right)=\frac{\partial T_{total}}{\partial \theta_{s}}=f\left(L_{0},\Delta x,R,K,\theta_{s}\right). \end{array}$$

**Figure 7:**
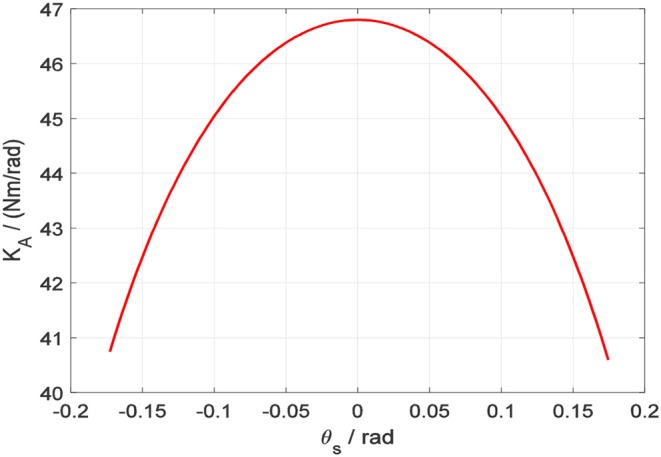
SEA rotary stiffness coefficient vs. four-spoke angular deflection.

The above equation expresses the non-linear relationship between the torque loaded on the four-spoke component and the four-spoke angular deflection. Based on the physical parameters of the SEA listed in Table 1, the fitted non-linear curve between rotary stiffness coefficient *K*_*A*_(θ_*s*_) and four-spoke angular deflection θ_*s*_ is presented in Figure 7.

**Table 1:** Geometric parameters of the SEA.

**Parameter**	***L*_**0**_ (mm)**	**Δ*x* (mm)**	***R* (mm)**	***K* (N/mm)**
Value	11	11	20	14.625

**Figure 8:**
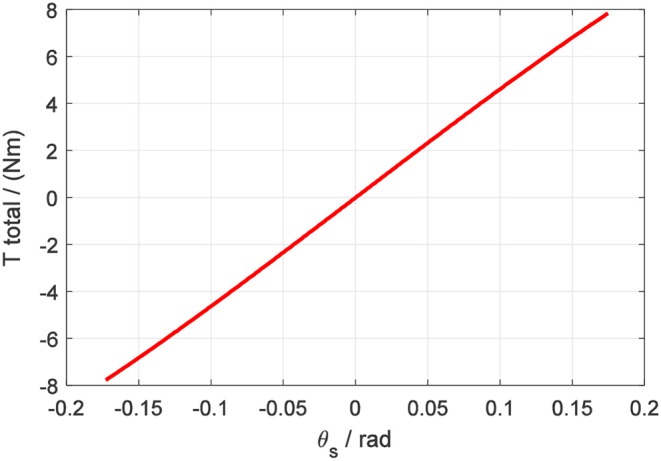
Torque on the four-spoke component vs. four-spoke angular deflection.

Correspondingly, the torque on the four-spoke can be calculated by the multiplication of *K*_*A*_ and θ_*s*_. In Figure 8, the fitted non-linear curve between output torsional torque *T*_*total*_ and four-spoke angular deflection θ_*s*_ is presented.

According to the above fitted non-linear curve, the output torque *T*_*total*_ of the elastic element has a continuous relationship with the angular deflection θ_*s*_. The non-linear function between *T*_*total*_ and θ_*s*_ can be simplified as

(9) $$\begin{array}{l} T_{total}=g\left(\theta_{s}\right) \end{array}$$

where *g*(θ_*s*_) is a continuous invertible function of θ_*s*_, which can be regarded as

(10) $$\begin{array}{l} g\left(\theta_{s}\right)=K_{A}\left(\theta_{s}\right)\theta_{s}. \end{array}$$

### The Transmission Model of Bowden Cable

**Figure 9:**
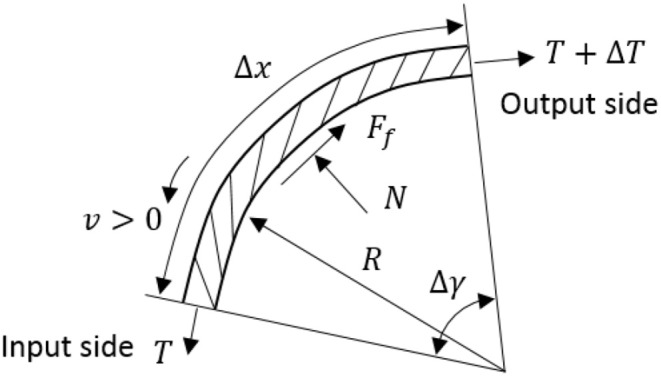
Forces diagram of the infinitesimal steel cable unit inside Bowden cable.

In this paper, the Bowden cable is used for energy and motion transmission, which is a common method for remote actuation (Kong et al., 2010; Asbeck et al., 2014). More specifically, the functionality of the pair of Bowden cables for each SEA is to transmit the output torque on SEA pulley to the corresponding DOF on the joint in a positive or negative direction. However, the introduction of the Bowden cable will cause some additional problems for the system, such as friction between steel cable and sheath, dead zone, and hysteresis, where friction is the most troublesome factor. Here, to compensate for the friction, the Bowden cable's transmission model is considered. Take an infinitesimal unit of the inner steel cable as a target, as shown in Figure 9, the forces and deflection diagram are presented below.

The friction of the steel cable unit results from the normal force, so the below equations can be obtained

(11) $$\begin{array}{l} F_{f}=\mu Nsign\left(v\right)=\{\begin{array}{l} \mu N,\;v\geq 0\\ -\mu N,\;v<0 \end{array} \end{array}$$

(12) $$\begin{array}{l} \Delta T=-F_{f} \end{array}$$

(13) $$\begin{array}{l} \varepsilon =\frac{\Delta L}{L}=\frac{1}{EA}T=\rho T \end{array}$$

(14) $$\begin{array}{l} \mu =\{\begin{array}{l} \mu_{s},\;v=0\\ \mu_{d},\;v\neq 0 \end{array} \end{array}$$

where *E*, *A*, and ε are Young's modulus, cross-area, and strain, respectively. *v* is the velocity of cable, and *T* is the tension force on the cable. *F*_*f*_ and *N* are the friction force and normal force acting on the cable, respectively. μ is the Column friction coefficient. Suppose there is a relative sliding between the inner cable and its sheath, and *v* has the same direction along the entire cable. Then the infinitesimal cable unit will satisfy the following equations

(15) $$\begin{array}{l} N=Td\gamma =T\frac{ds}{R} \end{array}$$

(16) $$\begin{array}{l} dT=-F_{f}=-\mu T\frac{ds}{R}sign\left(v\right) \end{array}$$

where *ds* is the arc length corresponding to the central angle *d*γ. *R* is the radius of the curvature of the steel cable unit.

Now, the above equations can be re-written in matrix form as follows

(17) $$\begin{array}{l} \left[\begin{array}{c} \frac{dT}{ds}\\ \frac{d\varepsilon }{ds} \end{array}\right]=\left[\begin{array}{cc} -\frac{\mu }{R}sign\left(v\right) & 0\\ \frac{1}{EA} & 0 \end{array}\right]\left[\begin{array}{c} T\\ \varepsilon \end{array}\right]+\left[\begin{array}{c} 0\\ -\frac{1}{EA} \end{array}\right]T_{0} \end{array}$$

where *T*_0_ is the pre-load tension on the steel cable. In the above augmented state-space equations, only the tension problem is addressed, and the solution of the tension differential equation is given as

(18) $$\begin{array}{l} T_{out}\left(s\right)=\{\begin{array}{l} T_{in}\exp\left[-\frac{\mu s}{R}sign\left(v\right)\right],\;s<L_{1}\\ \;T_{0},\;s\geq L_{1} \end{array} \end{array}$$

where *L*_1_ represents the length of the steel cable with displacement, which is a time-related variable. And it depends on the following conditions:

(1) When the input tension of the cable is between *T*_0_ to *T*_*in*_, the length of the cable with displacement is within 0 and *L*_1_.

(2) When the deformation of the cable exceeds *L*_1_, the tension of the cable does not increase but maintains a constant value.

We assume that as the input cable tension increases, *L*_1_ increases and eventually *L*_1_ becomes *L* (the length of the entire Bowden cable). From Equation (18), it is clear that *T*_*out*_ changes instantly with the change of *T*_*in*_. *T*_*out*_ will be loaded directly on the DOF to act as the input torque of the DOF. As designed, the radius of SEA output pulley equals to the radius of joint pulley. Therefore, the relationship between input tension *T*_*in*_ and output tension *T*_*out*_ on Bowden cable can be regarded as

(19) $$\begin{array}{l} {T_{out}=T}_{in}\exp\left[-\frac{\mu L}{R}sign\left(v\right)\right]. \end{array}$$

The above equation shows that if the radius of curvature remains constant, there is a linear function between *T*_*in*_ and *T*_*out*_. In the real Bowden cable system, since the friction force on Bowden cable is caused by the normal force, and only the tense cable could generate normal force between the inner cable and outer sheath. Therefore, no matter which direction of the cable input torque, the output torque of the Bowden cable end side is given as

(20) $$\begin{array}{l} T_{out}=T_{in}e^{-\mu \theta \left(L\right)},\;v>0. \end{array}$$

Based on the above equation, the transmission model of the Bowden cable only depends on the Column friction coefficient μ and the central angle θ(*L*) corresponding to the entire length of the Bowden cable.

### Dynamic Modeling of the Remote Actuation System

In the configuration of remote actuation, it is elaborately designed that the cable directions of the beginning side and the end side are the tangential directions of their corresponding pulleys. Also, the cable center axis and the sheath center axis are aligned. Therefore, no additional friction force caused by the misalignment exists during the energy and force transmission on the Bowden cable. The torque overcoming the friction between Bowden cable and outer sheath can be written as

(21) $$\begin{array}{l} f_{c}=\tau_{cin}-\tau_{cout} \end{array}$$

where τ_*cin*_ = *T*_*in*_*r*_1_ and τ_*cout*_ = *T*_*out*_*r*_2_ represent the equivalent output torque on SEA pulley and input torque on the joint pulley. Additionally, by neglecting the friction effect, the single SEA's dynamics can be expressed as

(22) $$\begin{array}{l} {\;J}_{in}\ddot{q}+D_{q}\dot{q}+\tau_{cin}=\tau_{e}\left(\theta_{s}\right)\\ J_{m}\ddot{\theta }+D_{\theta }\dot{\theta }+\tau_{e}\left(\theta_{s}\right)=\tau \\ \;g\left(\theta_{s}\right)=\tau_{e}\left(\theta_{s}\right) \end{array}$$

where *q* and θ are the SEA output pulley angular position and gear reducer shaft angular position, respectively. The four-spoke angular deflection is defined as θ_*s*_ = θ−*q*, and it can be measured directly through the rotary encoder fixed on the head of the SEA. *J*_*in*_ is the inertia of the inner sleeve, and *J*_*m*_is the inertias of servo motor with planetary gear reducer. *D*_*q*_ and *D*_θ_ are the viscous coefficients of the inner sleeve and the servo motor with reducer. τ_*cin*_ is the output torque of SEA pulley (at the same time input torque of Bowden cable starting side). τ and τ_*e*_(θ_*s*_) are the output torques from the motor reducer and the elastic element, respectively.

**Figure 10:**
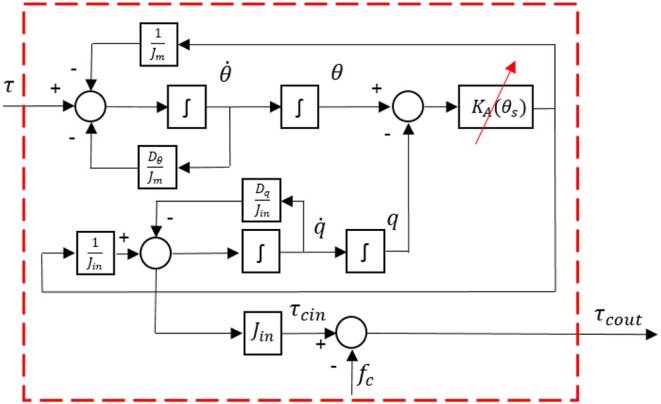
Dynamics diagram of the remote actuation method with SEA.

Furthermore, the dynamics of the system in Equations (21) and (22) are illustrated in Figure 10, where the input is the torque from reducer connected with the servo motor, and the output is the equivalent torque on the joint pulley.

## Controller Design

### Torque Control

The objective of the torque control is to design a closed-loop system and make the actual output torque on the end side of Bowden cable track the desired torque reference. By combining the equations in Equation (22), the following equation can be derived as

(23) $$\begin{array}{l} J_{m}\ddot{\theta }=\tau -D_{\theta }\dot{\theta }-J_{in}\ddot{q}-D_{q}\dot{q}-\tau_{cout}-f_{c}. \end{array}$$

The joint input torque τ_*cout*_ is the variable that needs to track the reference torque signal, which is also the feedback. By substituting (21) and (22) to (23), the following equation can be obtained corresponding to τ_*cout*_

(24) $$\begin{array}{l} {\ddot{\tau }}_{cout}=\frac{K_{A}}{J_{m}}\tau -\frac{D_{\theta }}{J_{m}}{\dot{\tau }}_{cout}-\frac{K_{A}}{J_{m}}\tau_{out}-J_{in}q^{\left(4\right)}\\ \;-\left(D_{q}+\frac{D_{\theta }}{J_{m}}J_{in}\right)q^{\left(3\right)}-\left(K_{A}+\frac{D_{\theta }D_{q}}{J_{m}}+\frac{K_{A}}{J_{m}}J_{in}\right)\ddot{q}\\ \;-\frac{K_{A}\left(D_{\theta }+D_{q}\right)}{J_{m}}\dot{q}-\frac{D_{\theta }}{J_{m}}\dot{f_{c}}-\frac{K_{A}}{J_{m}}f_{c}-\ddot{f_{c}} \end{array}$$

where the derivative of *q* with different orders reflects the motion of the joint. To control the input torque of the joint, we propose a complex controller containing four terms as described in Yu et al. (2015)

(25) $$\begin{array}{l} \tau =\tau_{h}+\tau_{f}+\tau_{d}+\tau_{fb} \end{array}$$

where τ_*h*_ is used to compensate for the error caused by the joint motion. τ_*f*_ is used to compensate for the error caused by friction. τ_*d*_ is used to eliminate external disturbance. τ_*fb*_ is the feedback term. Substitute (25) to (24) and the following equation is derived

(26) $$\begin{array}{l} {\ddot{\tau }}_{cout}=\frac{K_{A}}{J_{m}}\left(\tau_{h}+\tau_{f}+\tau_{d}+\tau_{fb}\right)-\frac{D_{\theta }}{J_{m}}{\dot{\tau }}_{cout}-\frac{K_{A}}{J_{m}}\tau_{out}-J_{in}q^{\left(4\right)}\\ \;-\left(D_{q}+\frac{D_{\theta }}{J_{m}}J_{in}\right)q^{\left(3\right)}-\left(K_{A}+\frac{D_{\theta }D_{q}}{J_{m}}+\frac{K_{A}}{J_{m}}J_{in}\right)\ddot{q}\\ \;-\frac{K_{A}\left(D_{\theta }+D_{q}\right)}{J_{m}}\dot{q}-\frac{D_{\theta }}{J_{m}}\dot{f_{c}}-\frac{K_{A}}{J_{m}}f_{c}-\ddot{f_{c}}. \end{array}$$

**Figure 11:**
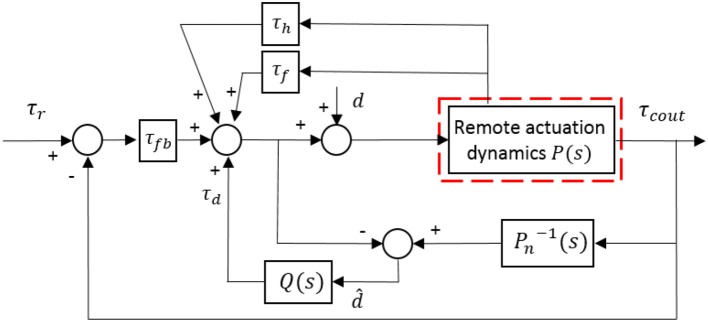
Block diagram of the proposed torque controller with motion compensation, friction compensation, feedback control, and DOB.

The block diagram of the torque controller for the remote actuation system is presented in Figure 11, where the red dashed line part represents the remote actuation dynamics, as shown in Figure 10. Then the next step is to determine the four terms in Equation (25) to simplify the closed-loop system.

**(1) Compensation for the joint motion**

The compensation term should include all factors associated with *q*, which can minimize the force between the human arm and exoskeleton robot during the joint movement. Then based on (26), the term is designed as

(27) $$\begin{array}{l} \tau_{h}=\frac{J_{m}}{K_{A}}J_{in}q^{\left(4\right)}+\left(\frac{J_{m}}{K_{A}}D_{q}+\frac{D_{\theta }}{K_{A}}J_{in}\right)q^{\left(3\right)}\\ \;+\left(J_{m}+\frac{D_{\theta }D_{q}}{K_{A}}+J_{in}\right)\ddot{q}+\left(D_{\theta }+D_{q}\right)\dot{q}. \end{array}$$

**(2) Compensation for the friction**

In the same way, the friction compensation term can be derived as

(28) $$\begin{array}{l} \tau_{f}=\frac{J_{m}}{K_{A}}\ddot{f_{c}}+\frac{D_{\theta }}{K_{A}}\dot{f_{c}}+f_{c}. \end{array}$$

**(3) Design of DOB**

Since the compensation of joint motion and friction may cause additional noise and error due to the high order time derivative terms, a DOB is designed for the system to deal with the noise and error. After substituting (27) and (28) to (26), the system (26) with remaining disturbance can be given as

(29) $$\begin{array}{l} {\ddot{\tau }}_{cout}=\frac{K_{A}}{J_{m}}\left(\tau_{d}+\tau_{fb}\right)-\frac{D_{\theta }}{J_{m}}{\dot{\tau }}_{cout}-\frac{K_{A}}{J_{m}}\tau_{out}+d \end{array}$$

where *d* is the unknown disturbance of the entire system.

An estimation of the actual disturbance is recruited to determine the expression of the DOB, which is represented as $\hat{d\;}$in Figure 11. Besides, *P*(*s*) is the actual system model, and *P*_*n*_(*s*) is the nominal model without any disturbance.

From Equation (29), the nominal model of the system can be expressed as

(30) $$\begin{array}{l} P_{n}=\frac{\frac{K_{A}}{J_{m}}}{s^{2}+\frac{D_{\theta }}{J_{m}}s+\frac{K_{A}}{J_{m}}}. \end{array}$$

Due to the inverse of *P*_*n*_ is not a rational function, a low-pass filter is added to make the multiplication between ${P_{n}}^{-1}$ and the filter implementable. The second-order low-pass filter can be selected as the following format

(31) $$\begin{array}{l} Q\left(s\right)=\frac{\delta_{2}}{s^{2}+\delta_{1}s+\delta_{2}} \end{array}$$

where the coefficients of δ_1_ and δ_2_ should be adjusted to satisfy the characteristic of disturbance suppression. Based on the above analysis, the DOB is given by

(32) $$\begin{array}{l} \tau_{d}=-\frac{\delta_{2}}{s^{2}+\delta_{1}s+\delta_{2}}\hat{d}. \end{array}$$

**(4) Feedback control**

Define the torque tracking error between the reference and actual values as *e*_1_(*t*) = τ_*r*_(*t*) − τ_*cout*_(*t*), and then define $e_{2}\left(t\right)={\dot{e}}_{1}\left(t\right)={\dot{\tau }}_{r}\left(t\right)-{\dot{\tau }}_{cout}\left(t\right)$, then the equation (29) can be written as

(33) $$\begin{array}{l} {\ddot{e}}_{1}={\ddot{\tau }}_{r}+\frac{K_{A}}{J_{m}}\tau_{r}+\frac{D_{\theta }}{J_{m}}{\dot{\tau }}_{r}-\frac{K_{A}}{J_{m}}\left(\tau_{d}+\tau_{fb}\right)-\frac{K_{A}}{J_{m}}e_{1}-\frac{D_{\theta }}{J_{m}}{\dot{e}}_{1}-d. \end{array}$$

The state vector is defined as $e={\left[e_{1},\;e_{2}\right]}^{T}$, then the state space equation of the differential equation can be given as

(34) $$\begin{array}{l} \;\dot{e}=A_{1}e-B_{1}\tau_{fb}+B_{1}\left(\frac{J_{m}}{K_{A}}{\ddot{\tau }}_{r}+\frac{D_{\theta }}{K_{A}}{\dot{\tau }}_{r}+\tau_{r}-\bar{d}\right)\\ {\;A}_{1}=\left[\begin{array}{cc} 0 & 1\\ -\frac{K_{A}}{J_{m}} & -\frac{D_{\theta }}{J_{m}} \end{array}\right],B_{1}=\left[\begin{array}{c} 0\\ \frac{K_{A}}{J_{m}} \end{array}\right],\bar{d}=\tau_{d}+\frac{J_{m}}{K_{A}}d. \end{array}$$

Therefore, the feedback control term is given by

(35) $$\begin{array}{l} \tau_{fb}=\frac{J_{m}}{K_{A}}{\ddot{\tau }}_{r}+\frac{D_{\theta }}{K_{A}}{\dot{\tau }}_{r}+\tau_{r}+Ke \end{array}$$

where *K* ∈ ℝ^1 × 2^ represents the feedback gain matrix.

### Impedance Control

**Figure 12:**
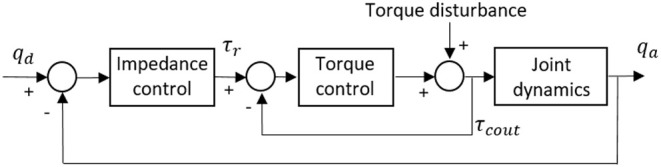
Impedance control diagram based on torque control.

Impedance control is not to control the position or force/torque directly, but to satisfy the dynamical correspondence between the force/torque and desired position through adjusting the impedance (Hogan, 1985). For the ULRR proposed in this paper, only impedance control in the joint space is considered. In other words, each actuated DOF has an individual impedance controller. The design of the impedance controller structure on each DOF remains the same, but with different gains, and the block diagram is shown in Figure 12. In order to illustrate the impedance controller design, the DOF of elbow flexion/extension is taken as an example.

In Figure 12, it is clear that there are two main loops, where the inner loop is the torque controller designed in the last section, while the outer loop is the impedance controller, which appears as a proportional-derivative (PD) position feedback controller. *q*_*d*_ and *q*_*a*_ represent the desired and actual angular trajectories of the targeted joint DOF, respectively. The external position feedback controller determines the desired input torque τ_*r*_, and the desired input torque can be given by

(36) $$\begin{array}{l} \tau_{r}\left(q_{a},{\dot{q}}_{a}\right)=K_{j}\left(q_{d}-q_{a}\right)+B_{j}\left({\dot{q}}_{d}-{\dot{q}}_{a}\right) \end{array}$$

where *K*_*j*_ and *B*_*j*_ represent the virtual stiffness and damping coefficients, respectively.

## Simulation Results

### Torque Control

**Figure 13:**
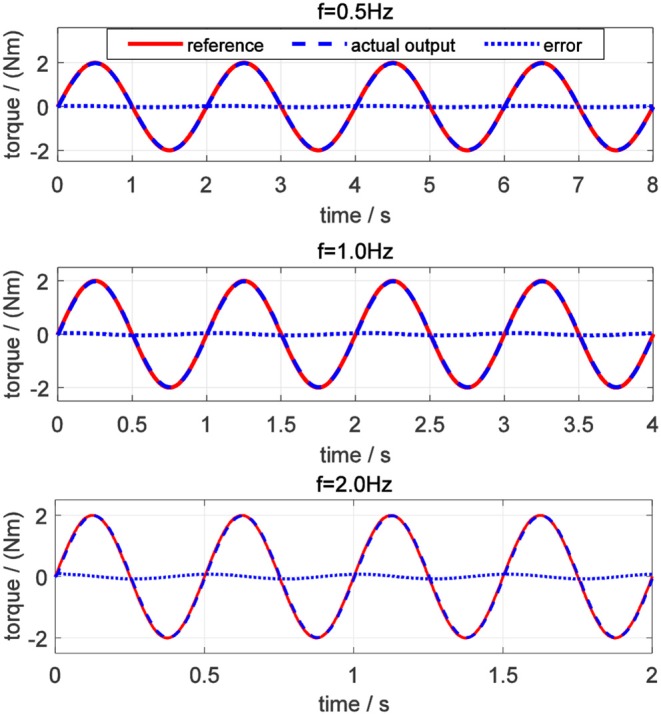
Torque tracking simulation results on elbow joint without disturbance.

For evaluating the torque controller, the simulations for tracking desired sinusoidal torque signals with different frequencies were performed on elbow flexion/extension DOF. Commonly, the maximum periodic motion frequency of the human elbow joint is 2 Hz, so the frequencies of desired torque trajectory were set to be 0.5, 1.0, and 2.0 Hz, respectively. Also, the amplitude of the sinusoidal signals was set to be 2.0 Nm for different frequencies. In Figure 13, the torque tracking simulation results without disturbance are presented, where the solid red line, dashed blue and dotted blue lines represent the desired torque, actual output torque, and torque tracking error, respectively.

**Figure 14:**
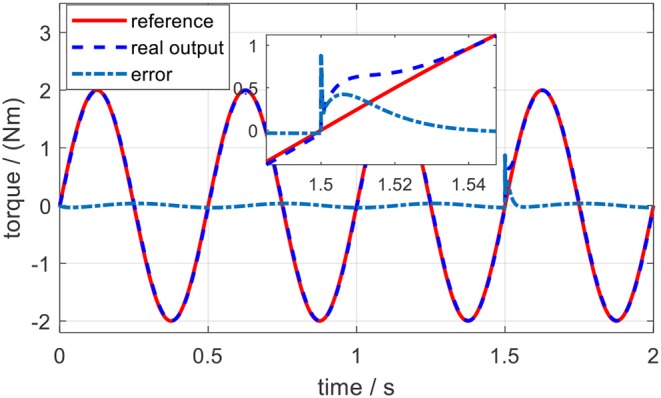
Torque tracking simulation results on the elbow exoskeleton with torque disturbance at 1.5 s.

The tracking performance at a low frequency is better than that at high frequency. Root mean square errors (RMSE) between the desired and actual torque for the three frequencies were calculated: 0.062, 0.118, and 0.197 Nm. In addition, the peak relative error was always <5%, which shows that the proposed torque controller can achieve excellent fidelity while tracking the desired torque. To simulate the external disturbance, a constant torque signal with an amplitude of 1.0 Nm was added into the closed-loop system at 1.5 s. Take the desired torque signal with 2 Hz in Figure 13 as an example, and the torque tracking simulation result with disturbance is shown in Figure 14. The result shows that the proposed torque controller can effectively eliminate the external torque disturbance and force the tracking error back to the level before the disturbance within a duration of 0.04 s.

### Impedance Control

**Figure 15:**
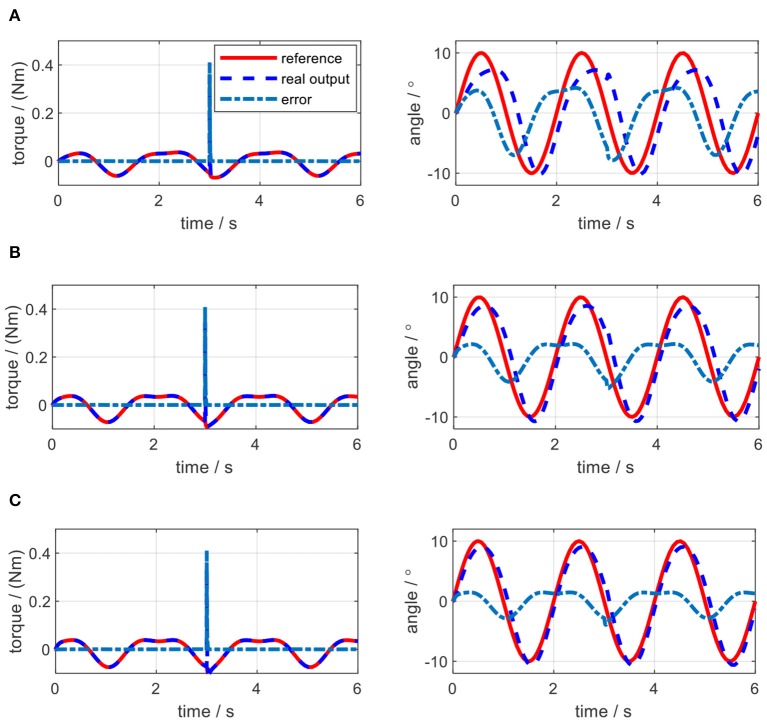
Impedance control results of the elbow exoskeleton with a sinusoidal reference joint trajectory and a constant external torque disturbance at 3.0 s. **(A)** *K*_*j*_ = 0.5 Nm/rad, *B*_*j*_ = 0 Nms/rad. **(B)** *K*_*j*_ = 1.0 Nm/rad, *B*_*j*_ = 0 Nms/rad. **(C)** *K*_*j*_ = 1.5 Nm/rad, *B*_*j*_ = 0 Nms/rad.

The impedance controller in this paper is used to switch the exoskeleton working mode between human-in-charge and robot-in-charge by adjusting both the virtual stiffness and damping coefficients. As can be seen in Figure 12, to implement impedance control, the desired angular trajectory on the elbow joint was designed as a sinusoidal signal with an amplitude of 10 and a frequency of 0.5 Hz. Similarly, a constant external torque disturbance with an amplitude of 0.5 Nm was also applied to the elbow joint at 3.0 s. By adjusting the virtual stiffness coefficient, the impedance control results are shown in Figure 15. The tiny influence of the virtual damping coefficient changing from 0 to 0.01 Nms/rad on the results of impedance control can be neglected, so no such results are presented here.

As shown in Figure 15A, when the virtual stiffness is at a small level, the elbow joint trajectory tracking error is relatively high, especially around 3 s due to the induce of torque disturbance. But after 3 s, the tracking error is forced back to the level before the disturbance time point by applying the impedance controller. With the increase of the coefficient *K*_*j*_, the elbow joint tracking error is reduced and the capability of resisting torque disturbance is enhanced. In Figure 15, with the increase of *K*_*j*_, the RMSE between the actual joint angle and reference are 3.85, 2.26, and 1.52, degrees respectively. However, Figure 15 shows that there is no significant change for the torque tracking performance due to the increase of *K*_*j*_. Although the high impedance coefficients, especially high virtual stiffness coefficient *K*_*j*_, are beneficial for external disturbance resistance and angle trajectory tracking performance; they could limit interaction adaptability between the robotic device and human wearer. Therefore, two assistive patterns based on the impedance control are established, including human in-charge control (low impedance coefficients) and robot in-charge control (high impedance coefficients). In the clinical application, these two assistive patterns can be customized according to the rehabilitation stages of the patients.

## Experimental Results and Discussions

### Validation of the Mathematical Model

**Figure 16:**
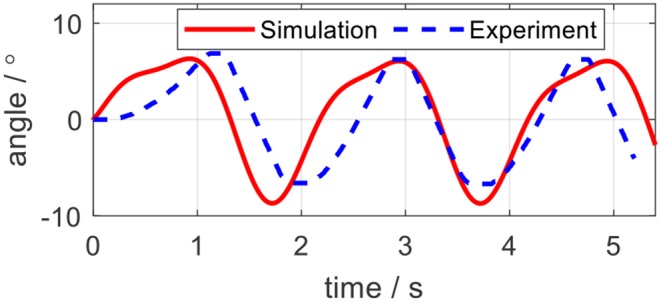
Analytical and experimental angle trajectories on the elbow exoskeleton in the open-loop test.

The aforementioned modeling and simulation sections are based on the mathematical model (22). To solidify the correctness of the mathematical model, an open-loop experiment on the elbow testbed was performed. Instead of defining a random input torque for the test, the input torque was calculated based on (36) offline by using the same desired elbow joint trajectory and setting *K*_*j*_ = 0.3 Nm/rad, *B*_*j*_ = 0 Nms/rad. The computed torque was applied on both the physical elbow exoskeleton and the mathematical model for evaluation. The joint angle trajectories in the elbow joint and the simulation are shown in Figure 16. The solid red line represents the analytical simulation, while the dashed blue line represents the real angular position of the elbow joint. The results indicate that although the amplitudes and frequencies of two trajectories are similar, there exists a phase shift for the experimental results, as well as time delay during the movement starting period. The RMSE is 4.06, degrees and the correlation coefficient (CC) between these two trajectories is 0.82.

One possible reason for the trajectory difference is the modeling uncertainties for the force transmission model in Equation (20). Since we did not consider the hysteresis of the Bowden cable in the simulation, when implementing the same input, the physical position would lag behind the simulation output. In addition, we hypothesize that there exist constant friction coefficient μ and constant central angle θ(*L*) for Bowden cable, however, it exhibits non-isotropic μ and θ(*L*) along the length of the Bowden cable. Therefore, only from open-loop, it would be difficult to accurately track a specific trajectory. The closed-loop position controller is required, which is referred impedance controller in this paper. The evaluation of the closed-loop system with the impedance controller is given in the subsequent section.

### Torque Control

**Figure 17:**
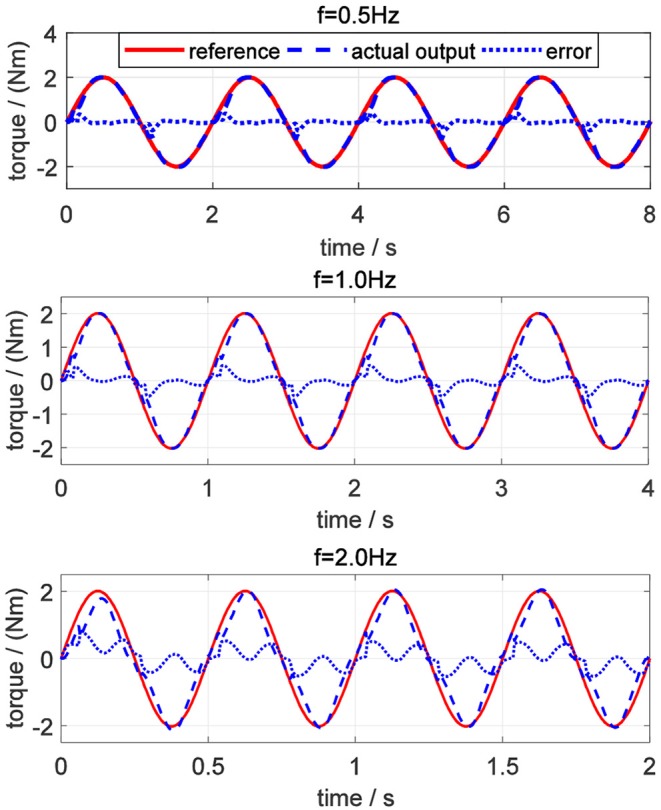
Torque tracking experimental results on the elbow exoskeleton without torque disturbance.

Like in simulation, the desired sinusoidal torque signals with the same amplitude and frequencies were utilized to test the torque controller on the elbow joint platform. The experimental results to track desired sinusoidal torque signals without external torque disturbance are shown in Figure 17. The average error, average relative error, peak error, and peak relative error between the desired and actual torque signals with different frequencies are listed in Table 2. The results show that the torque tracking error is sensitive to the frequency of the sinusoidal torque signals, and with the increasing of the frequency, the tracking performance becomes worse, which corresponds to the results in the simulation. When the frequency of the desired sinusoidal torque signal is <1.0 Hz, the tracking performance is acceptable, with an average relative error of <10%.

**Table 2:** Torque tracking error for references with different frequencies in experiments.

**Frequency/Hz**	**Average error (N·m)**	**Average relative error (%)**	**Peak error (N·m)**	**Peak relative error (%)**
0.5	0.078	3.9	0.382	19.1
1.0	0.130	6.5	0.454	22.7
2.0	0.250	12.5	0.494	24.7

**Figure 18:**
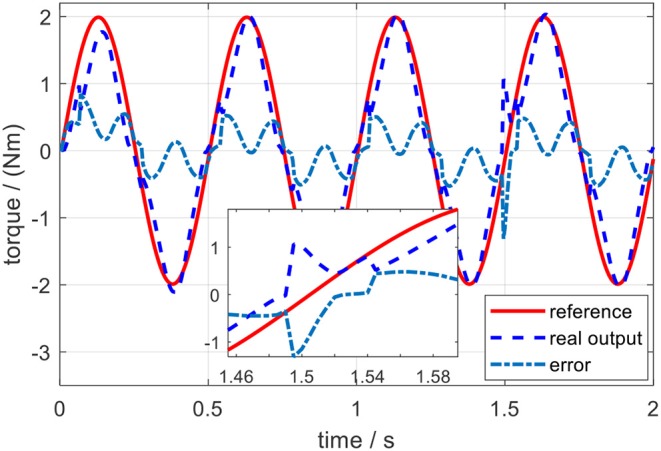
Torque tracking experimental results of a sinusoidal signal of 2 Hz on the elbow exoskeleton with torque disturbance around 1.5 s.

The same external torque disturbance mentioned in section Torque Control was added into the closed-loop elbow exoskeleton system around 1.5 s. Similarly, take the desired torque signal with 2 Hz in Figure 17 as an example, and the torque tracking experimental result with external torque disturbance is shown in Figure 18. The result shows that the proposed torque controller can also effectively eliminate the external torque disturbance and force the tracking error back to the level before the disturbance within a duration of 0.05 s.

### Impedance Control

**Figure 19:**
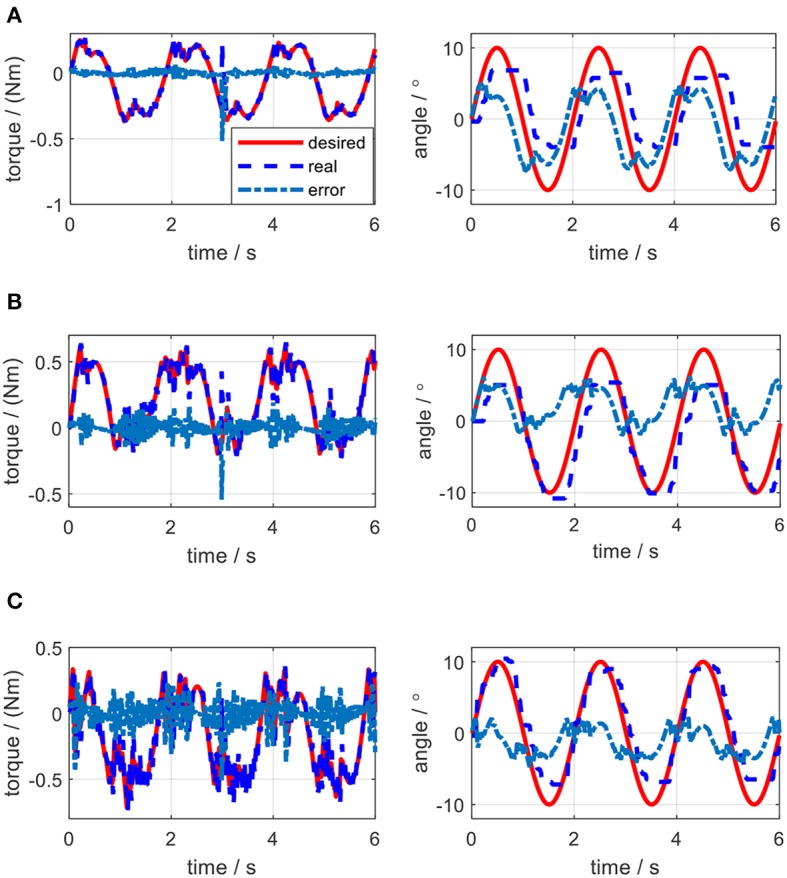
Experimental results of impedance control on the elbow by changing stiffness coefficient. **(A)** *K*_*j*_=0.5 Nm/rad, *B*_*j*_ = 0 Nms/rad. **(B)** *K*_*j*_=1.0 Nm/rad, *B*_*j*_ = 0 Nms/rad. **(C)** *K*_*j*_=1.5 Nm/rad, *B*_*j*_ = 0 Nms/rad.

**Figure 20:**
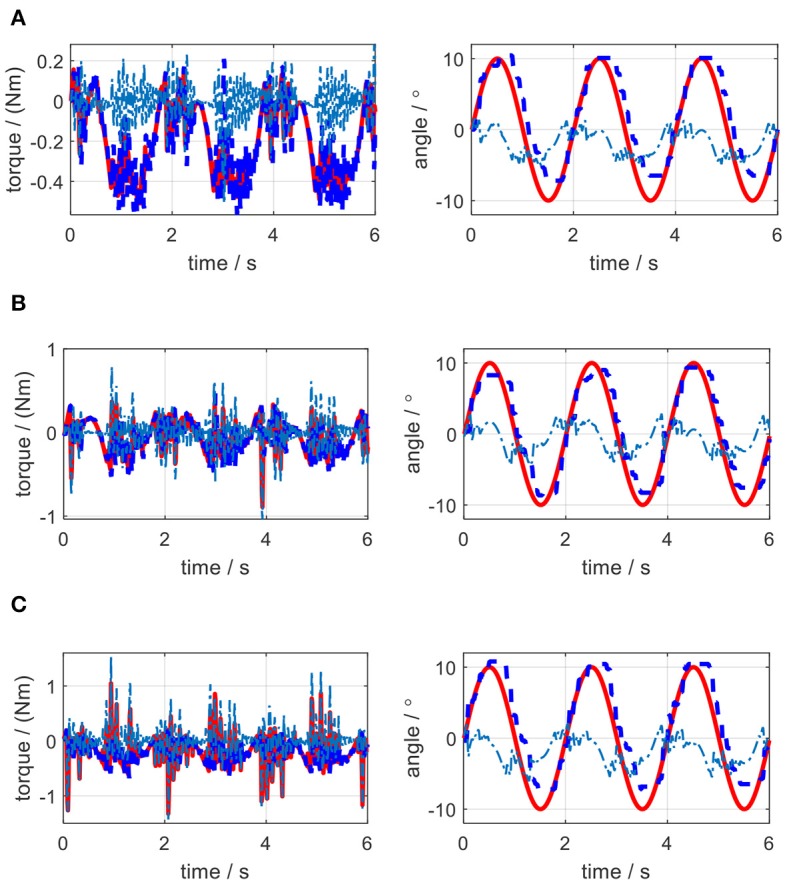
Experimental results of impedance control on the elbow by changing the damping coefficient. **(A)** *K*_*j*_=1.0 Nm/rad, *B*_*j*_ = 0.001 Nms/rad. **(B)** *K*_*j*_=1.0 Nm/rad, *B*_*j*_ = 0.005 Nms/rad. **(C)** *K*_*j*_=1.0 Nm/rad, *B*_*j*_ = 0.01 Nms/rad.

In the impedance control experiments, the desired trajectory for the elbow joint was set as the same sinusoidal single defined in the simulation. The initial angular position of the elbow joint was set at the middle point of the joint movement range. Different trials were operated by changing the values of *K*_*j*_ and *B*_*j*_. For each trial, the actual elbow joint trajectory was measured by the joint encoder, the desired torque on elbow joint pulley was calculated by using (36), and the actual torque loaded on the joint pulley were calculated based on the remote actuation system dynamics. By applying the same external torque disturbance described in section Impedance Control, the experimental results of torque tracking and joint trajectory tracking concerning each pair of *K*_*j*_ and *B*_*j*_ in impedance control are shown in Figures 19, 20, where the solid red lines, dashed blue lines, and centered blue lines represent the desired signals, actual output signals, and tracking errors.

**Figure 21:**
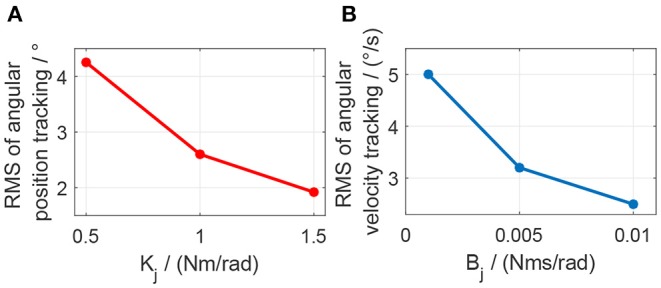
Effect of stiffness or damping coefficients change on the elbow joint angular position tracking or angular velocity tracking. **(A)** Change of stiffness coefficient. **(B)** Change of damping coefficient.

The elbow joint with zero impedance (both stiffness and damping coefficients are set to 0) is the pure human in-charge control mode and extremely compliant, which has the least disturbance resistance capability. The tests on *K*_*j*_ and *B*_*j*_ are separated into two sections. Firstly, as shown in Figures 19A–C, *B*_*j*_ is set to 0 Nms/rad all the time, while *K*_*j*_ is set to 0.5, 1.0, and 1.5 Nm/rad individually. The results show that with the increase of *K*_*j*_, although the joint trajectory tracking performance is improved, oscillation with high frequency occurs in the desired torque on the elbow joint. Besides, the torque tracking performance is deteriorated by the increased *K*_*j*_. The RMSE between the desired and actual elbow joint trajectories is decreased with the stiffness coefficient increase, which is shown in Figure 21A. However, when *K*_*j*_ continues increasing after 1.5 Nm/rad, the oscillation frequency of the desired torque on the elbow joint is too high, so the performance of the torque tracking becomes much worse due to the limited torque control bandwidth. Figures 19A,B also shows that the external torque disturbance around 3.0 s is addressed by the proposed torque controller, and the torque tracking error is forced back to the level before applying the disturbance. The short time duration to address the disturbance enables the stable joint trajectory tracking, so there is no significant angle change at 3.0 s for those three situations.

Secondly, as shown in Figures 20A–C, *K*_*j*_ is set to 1.0 Nm/rad, while *B*_*j*_ is set to 0.001, 0.005, and 0.01 Nms/rad individually. The results show that with the increase of *B*_*j*_, although there is no obvious change for the elbow joint trajectory tracking performance, the oscillation frequency and amplitude in the desired torque are both increasing, which results in the bad desired torque tracking performance, as shown in Figures 20B,C. However, the joint angular velocity tracking performance is improved with the increase of the damping coefficient, which is shown in Figure 21B that the RMS error between the desired and actual joint angular velocities is decreased.

## Discussions

A novel cable-driven remote actuation approach consisting of SEA and Bowden cables was proposed in this paper, which was applied in the ULRR design with four powered DOFs. The torque controller, composed of joint motion compensation, friction compensation, feedback, and DOB terms, was designed to test the practicability of this remote actuation method. Considering the torque controller as the inner loop, the impedance controller was designed by adding the PD-like outer loop. The torque tracking performance was validated on the ULRR elbow joint both in the simulation and experiments, and the effect of impedance coefficients on torque tracking and joint trajectory tracking were investigated on the elbow joint both in the simulation and experiments.

In the validation of the proposed torque controller, the results showed that the tracking error was sensitive to the frequency of the desired torque, and the tracking error was positively related to the frequency. Under the same desired torque, the torque tracking error in the simulation was less than that in experiments, as shown in Figures 13, 17. The possible reason is that for the real physical system, the modeling is much more complicated than the simplified simulation model. Although the proposed torque controller was robust to external torque disturbances, as shown in Figures 14, 18, it could not deal with the system modeling uncertainties. Recent studies Sun et al. (2019) and Yang et al. (2019) provide significant potentials to address unmodeled system dynamics, like input saturation and input delay, by using neural networks and integral terms.

In the validation of the impedance controller, both simulation and experimental results showed the change of impedance coefficients would affect the torque and elbow joint trajectory tracking. To mimic the external disturbance on the elbow joint, the constant external torque signal with a certain amplitude and at a certain time point was loaded on the elbow joint in both the simulation and experiments. The elbow joint was positioned to rotate in the horizontal plane, so the gravitational effect from the forearm was not considered in this work. With the increase of stiffness coefficient, the joint trajectory tracking error was decreased both in the simulation and experiments, while with the increase of damping coefficient, there was no clear change for the tracking error. However, during experiments, the stiffness or damping coefficients should not be set too large, because the large coefficients introduced desired torque oscillation with high frequency, and the proposed torque controller performance was deteriorated due to high signal frequency, as shown in Figures 19C, 20B,C.

As discussed in section Validation of the Mathematical Model, the accuracy of the mathematical model was quantified by the open-loop test. After presenting the results from the closed-loop system (with the impedance controller), the quantification results of the accuracy between the desired and actual trajectories in both simulation and experiments are listed in Table 3.

**Table 3:** Quantification results of the closed-loop system in simulation and experiment.

**Impedance coefficients**	**Closed-loop system in simulations**		**Closed-loop system in experiments**	
	**RMSE**	**CC**	**RMSE**	**CC**
K_*j*_ = 0.5 Nm/rad B_*j*_ = 0 Nms/rad	3.85°	0.85	4.28°	0.66
K_*j*_ = 1.0 Nm/rad B_*j*_ = 0 Nms/rad	2.26°	0.95	2.62°	0.89
K_*j*_ = 1.5 Nm/rad B_*j*_ = 0 Nms/rad	1.52°	0.99	1.95°	0.93

When *K*_*j*_ was set as a small value, like 0.5 Nm/rad, the experimental trajectory tracking performance of the closed-loop system was not as good as the open-loop system. But with the increase of *K*_*j*_, the experimental trajectory tracking performance of the closed-loop system was better than the open-loop system. However, in simulation, the trajectory tracking performance of the closed-loop system was better than the open-loop system for the three sets of impedance coefficients we selected.

From the results of impedance control, although we only focused on the elbow joint of the ULRR, without loss of generality, the interaction between human wearers and the ULRR can be categorized into two assistive patterns, human in-charge control, and robot in-charge control. To avoid oscillation, the damping coefficient should not be set too large. Furthermore, according to the rehabilitation stages of the patients, the assistive ratio could be set from zero to one corresponding to human in-charge control and robot in-charge control mode. Therefore, the design ULRR could be customized based on different clinical requirements. However, there also exist several limitations in the current work. For example, some modeling uncertainties were not considered, like the hysteresis of the Bowden cable when building the power transmission model and the non-isotropic μ and θ(*L*) along the length of the Bowden cable, as shown in Figure 16. Furthermore, although in simulation, shown in Figure 15, the effect of damping coefficient modulation to the impedance control performance was neglected, the noisy joint angular position signals along with the first-order time derivative signals (angular velocity) in the impedance control experiments limited the damping coefficient modulation as shown in Figure 20, where the higher damping severely deteriorated the torque tracking performance.

## Conclusion

In this paper, a novel cable-driven rotary series elastic actuator (SEA) was proposed to implement remote actuation. First, the new structural configuration of the novel remote actuator and its work mechanism were presented, after which the implementation of the upper limb rehabilitation robot was introduced. Based on the dynamical model of this remote actuation system, the torque controller with joint motion and friction compensation, PD feedback, and disturbance observer (DOB) terms were proposed. The impedance controller was also proposed to test the remote actuation's ability of disturbance resistance. Finally, the performance of both the torque and impedance controllers were verified in simulation and experiments. The results showed that this novel SEA with Bowden cable could achieve stable actuation for long-distance, which can be customized to meet the requirements of a wide range of implementations. In the future, we will focus on the impedance control of the full ULRR with four DOFs. Also, due to the non-linearity in the ULRR system, more advanced non-linear controllers need to be developed for better implementations.

## Data Availability Statement

The raw data supporting the conclusions of this article will be made available by the authors, without undue reservation, to any qualified researcher.

## Author Contributions

QZ contributed to the modeling, controller design, and simulation results of SEA. DS and WQ contributed to help with the experimental results. ZG and XX contributed to the design concept and writing.

### Conflict of Interest

The authors declare that the research was conducted in the absence of any commercial or financial relationships that could be construed as a potential conflict of interest.
